# Association between insulin resistance and coronary microcirculatory function and adverse cardiovascular events after PCI in non-diabetic STEMI patients

**DOI:** 10.3389/fcvm.2025.1657220

**Published:** 2026-01-05

**Authors:** Jinrong Zhong, Liming Tang, Yuanfeng Zhang

**Affiliations:** 1 Department of General Practice, Longyan First Affiliated Hospital of Fujian Medical University, Longyan, Fujian, China; 2 Sterilization Supply Center, Longyan First Affiliated Hospital of Fujian Medical University, Longyan, Fujian, China

**Keywords:** insulin resistance, Homeostasis Model Assessment of Insulin Resistance (HOMA-IR), coronary microcirculation, major adverse cardiovascular events, ST-segment elevation myocardial infarction

## Abstract

**Objective:**

Hyperglycemia and insulin resistance (IR) are common in patients with acute ST-segment elevation myocardial infarction (STEMI). However, the impact of IR on coronary microcirculatory function in non-diabetic STEMI patients remains unclear. This study aimed to investigate the relationship between IR and coronary microcirculation function and the incidence of major adverse cardiovascular events (MACE) at one year after Percutaneous Coronary Intervention (PCI) in non-diabetic STEMI patients.

**Methods:**

A total of 298 non-diabetic STEMI patients who underwent emergency PCI between 2022 and 2024 were retrospectively enrolled. Patients were divided into low, medium, and high groups based on Homeostasis Model Assessment of Insulin Resistance (HOMA-IR tertiles). Coronary microcirculation function was assessed using the index of microcirculatory resistance (IMR) and coronary flow reserve (CFR). IMR, CFR, and MACE were compared among the three groups. Pearson correlation was used to analyze the relationships between HOMA-IR and IMR/CFR. Logistic regression was used to identify predictors of microcirculatory dysfunction, and Cox regression was used to assess risk factors for MACE.

**Results:**

IMR increased and CFR decreased with rising HOMA-IR levels (*P* < 0.001). HOMA-IR was positively correlated with IMR and negatively correlated with CFR. Patients with higher HOMA-IR levels had significantly higher 1-year MACE incidence than those with lower HOMA-IR. Multivariate logistic regression analysis showed that high HOMA-IR was an independent predictor of elevated IMR (≥25) and reduced CFR (<2.0). Multivariate Cox regression analysis indicated that high HOMA-IR was an independent risk factor for MACE.

**Conclusion:**

In non-diabetic STEMI patients, elevated HOMA-IR is closely associated with coronary microcirculatory dysfunction and increased risk of 1-year MACE. Routine assessment of HOMA-IR may help identify high-risk individuals and support the development of individualized treatment strategies.

## 1 Introduction

Acute ST-segment elevation myocardial infarction (STEMI) is a severe form of acute coronary syndrome characterized by rapid onset and high mortality. Emergency percutaneous coronary intervention (PCI) has been widely adopted to reduce the extent of myocardial necrosis and improve both short- and long-term outcomes (1–4). However, even after successful recanalization of the infarct-related artery, a substantial proportion of patients still experience coronary microcirculatory dysfunction post-PCI. This is often manifested as poor myocardial perfusion or the no-reflow phenomenon, which significantly compromises therapeutic efficacy (5–7). Coronary microvascular dysfunction is not only a critical pathophysiological mechanism but has also been strongly associated with adverse cardiovascular events (MACE) following PCI (8).

In recent years, studies have shown that insulin resistance (IR) is prevalent not only in diabetic populations but also in non-diabetic patients with acute myocardial infarction. Moreover, the underlying metabolic disturbances associated with IR may exacerbate ischemia-reperfusion injury and further impair microvascular function (9–11). Although IR is common among STEMI patients, its impact on coronary microcirculation and clinical outcomes has not been thoroughly studied. Particularly in non-diabetic individuals, the clinical significance of “latent IR” has been underrecognized, underscoring the need for further exploration through both mechanistic and retrospective clinical investigations (12–14).

It is worth noting that although some studies suggest that IR may affect coronary perfusion and myocardial repair through various mechanisms (15), systematic research on the relationship between IR, microcirculatory function, and clinical prognosis in non-diabetic STEMI patients remains limited. This study addresses this gap with several innovative aspects. First, it focuses specifically on non-diabetic STEMI patients, thereby eliminating confounding effects of diabetes and its treatments on insulin metabolism and vascular function, and allowing a more accurate assessment of true IR as reflected by the Homeostasis Model Assessment of Insulin Resistance (HOMA-IR) index. Second, the study uses the index of microcirculatory resistance (IMR) and coronary flow reserve (CFR) for evaluation, which are more sensitive and quantitative than traditional TIMI flow grading, providing a more precise assessment of microcirculatory perfusion post-PCI. Third, by examining both immediate microcirculatory function and 1-year MACE during follow-up, the study offers a comprehensive view of the short-term pathophysiology and long-term clinical outcomes associated with IR, adding practical clinical relevance.

Therefore, this study retrospectively enrolled non-diabetic STEMI patients who underwent emergency PCI between 2022 and 2024. IR status was assessed using the HOMA-IR index. We investigated the association between IR and post-PCI coronary microcirculation (as measured by IMR and CFR), as well as 1-year MACE. Multivariate logistic regression and Cox proportional hazards models were used to determine whether IR is an independent predictor of microvascular dysfunction and adverse cardiovascular outcomes, with the aim of providing clinical evidence for early risk stratification and individualized intervention strategies in this patient population.

## 2 Methods

### 2.1 Study design and participants

This was a single-center, retrospective cohort study. A total of 298 hospitalized patients diagnosed with STEMI who underwent emergency PCI at our hospital between January 2022 and December 2024 were enrolled.

Inclusion criteria: (1) STEMI was diagnosed by trained clinicians according to the Fourth Universal Definition of Myocardial Infarction; (2) completion of emergency PCI within 24 h of admission; (3) Fasting blood glucose and insulin measured within 48 h after admission were used to calculate the HOMA-IR index, minimizing the influence of stress hyperglycemia; (4) All patients were non-diabetic, with no history of diabetes or use of hypoglycemic agents; abnormal blood glucose (ABG) was defined as fasting glucose ≥7.0 mmol/L or random glucose ≥11.1 mmol/L according to the Fourth Universal Definition of Myocardial Infarction.

Exclusion criteria: (1) a prior diagnosis of diabetes or HbA1c ≥ 6.5%; (2) chronic inflammatory disease, malignancy, or renal insufficiency (eGFR < 30 mL/min·1.73 m^2^, calculated by the CKD-EPI equation); (3) Cardiogenic shock or need for mechanical circulatory support; prior myocardial infarction (MI), PCI, or CABG; emergency PCI for NSTEMI or variant angina; or unstable angina and elective angiography for stable CAD were excluded to ensure a homogeneous population of first-onset STEMI patients treated with emergency PCI; (4) incomplete clinical data or loss to follow-up.

Patients were stratified into three groups based on admission HOMA-IR (Homeostasis Model Assessment of Insulin Resistance) tertiles: low HOMA-IR group, medium HOMA-IR group, and high HOMA-IR group.

This study was conducted in accordance with the ethical principles of the Declaration of Helsinki and its amendments and was approved by the institutional ethics committee. All data were anonymized and used solely for academic research. The original data were obtained from the hospital's electronic medical records and coronary physiology measurement platform.

### 2.2 Measurement of variables and grouping

Fasting blood glucose (FBG) and fasting insulin (FINS) data were retrieved retrospectively from the hospital's electronic laboratory records. All tests had been performed as part of routine clinical assessments within 48 h of admission, according to standardized institutional laboratory procedures. Fasting venous blood samples, obtained the morning after admission following the hospital's standard fasting protocol (≥8 h), were used for laboratory analyses. Glucose and insulin were measured in the hospital's central laboratory using automated biochemical analyzers, and the results were expressed in mmol/L and μU/mL, respectively.

Values considered implausible (e.g., due to data entry or assay errors) were verified against original laboratory reports by two independent investigators before inclusion. The HOMA-IR index was calculated using the formula: The HOMA-IR index was calculated using the formula: HOMA-IR = FINS (μU/mL) × FBG (mmol/L)/22.5.

Immediately after PCI, coronary microcirculatory function was assessed using a pressure/temperature sensor-tipped guidewire (PressureWire X, Abbott, USA). The following parameters were measured: Index of Microcirculatory Resistance (IMR): calculated as distal coronary pressure multiplied by mean transit time; IMR ≥ 25 was defined as elevated microvascular resistance. Coronary Flow Reserve (CFR): defined as the ratio of hyperemic to baseline coronary blood flow; CFR < 2.0 was considered reduced microvascular reserve. All assessments were performed immediately after PCI by experienced technicians using a standard adenosine-induced hyperemia protocol.

### 2.3 Follow-up and observational indicators

All patients were followed for at least one year. Follow-up data were collected via outpatient visits or telephone interviews. MACE included all-cause death, recurrent myocardial infarction, rehospitalization for heart failure, and target vessel revascularization. The first occurrence of any of these events was recorded as MACE.

Primary study endpoints included: immediate post-PCI IMR and CFR values; presence of microcirculatory dysfunction (IMR ≥ 25 and/or CFR < 2.0); and incidence of MACE within one year.

### 2.4 Statistical analysis

All statistical analyses were performed using SPSS version 26.0 (IBM Corporation, USA). Continuous variables were tested for normality and expressed as mean ± standard deviation. One-way analysis of variance (ANOVA) was used for comparison among the three groups, with Bonferroni correction for pairwise comparisons. Categorical variables were presented as counts and percentages and compared using the chi-square test or Fisher's exact test as appropriate. Pearson correlation coefficients were used to assess linear relationships between HOMA-IR and IMR/CFR. Multivariate logistic regression was used to evaluate the predictive value of HOMA-IR for microcirculatory dysfunction (defined as IMR ≥ 25 and/or CFR < 2.0), with variables selected based on clinical relevance and univariate analysis results. Cox proportional hazards models were used to analyze the relationships between HOMA-IR, IMR, CFR, and 1-year MACE, with hazard ratios (HRs) and 95% confidence intervals (CIs) calculated after adjustment for potential confounders. A two-tailed *P* value < 0.05 was considered statistically significant.

## 3 Results

### 3.1 Baseline characteristics across HOMA-IR tertiles

A total of 298 non-diabetic STEMI patients were included in this study and divided into three groups based on HOMA-IR tertiles: low (*n* = 99), medium (*n* = 100), and high (*n* = 99). There were no statistically significant differences among the three groups in terms of age, sex, BMI, smoking history, hypertension, admission glucose, HbA1c, lipid profile, creatinine, or left ventricular ejection fraction (LVEF) (all *P* > 0.05), indicating good comparability across groups (Table 1).

**Table 1:** Baseline characteristics Among HOMA-IR groups.

Variable	Low HOMA-IR (*n* = 99)	Medium HOMA-IR (*n* = 100)	High HOMA-IR (*n* = 99)	F/*χ*^2^	*P*-value
Age (years)	60.2	61.4	62.1	0.80	0.426
Male (%)	72 (72.7%)	74 (74.0%)	75 (75.8%)	0.25	0.882
BMI (kg/m^2^)	24.8	25.1	25.6	0.83	0.412
Smoking history (%)	51 (51.5%)	55 (55.0%)	56 (56.6%)	0.46	0.633
Hypertension (%)	46 (46.5%)	50 (50.0%)	52 (52.5%)	0.62	0.537
Admission glucose (mmol/L)	6.1	6.3	6.5	1.08	0.281
HbA1c（%）	5.7	5.8	5.9	0.89	0.372
Total cholesterol (mmol/L)	4.3	4.5	4.6	0.97	0.334
LDL-C (mmol/L)	2.6	2.7	2.8	1.06	0.291
Creatinine (μmol/L)	83.2	85.1	86.7	0.82	0.439
LVEF (%)	53.4	52.8	52.2	0.73	0.468

Data are presented as means for continuous variables or counts (percentages) for categorical variables. HOMA-IR, Homeostasis Model Assessment of Insulin Resistance; BMI, body mass index; HbA1c, glycated hemoglobin; LDL-C, Low-density lipoprotein cholesterol; LVEF, Left ventricular ejection fraction. Comparisons among the three HOMA-IR groups were performed using one-way analysis of variance (ANOVA) for continuous variables and chi-square (χ^2^) tests for categorical variables. *P*  <  0.05 was considered statistically significant.

### 3.2 Coronary microcirculatory function by HOMA-IR level

To explore the effect of insulin resistance on coronary microcirculatory function, we compared post-PCI IMR and CFR values among the three groups. IMR was significantly higher, and CFR significantly lower, in the high HOMA-IR group (*P* < 0.001 for both) (Table 2). Bonferroni *post hoc* comparisons revealed that IMR was significantly higher in the high HOMA-IR group than in the medium (*P* = 0.004) and low groups (*P* < 0.001). Similarly, CFR was significantly lower in the high group than in the other two (both *P* < 0.001), suggesting that higher IR is associated with increased microvascular resistance and decreased coronary flow reserve.

**Table 2:** Coronary microcirculatory function Among HOMA-IR groups.

Indicator	Low HOMA-IR (*n* = 99)	Medium HOMA-IR (*n* = 100)	High HOMA-IR (*n* = 99)	*F*-value	*P*-value	Bonferroni comparison
IMR	21.8 ± 7.2	25.7 ± 8.4	29.6 ± 9.1	14.89	<0.001	High > Medium (*P* = 0.004); High > Low (*P* < 0.001)
CFR	21.8 ± 7.2	2.39 ± 0.51	2.01 ± 0.49	22.17	<0.001	High < Medium (*P* < 0.001); High < Low (*P* < 0.001)

Data are expressed as mean  ±  standard deviation. IMR, Index of microcirculatory resistance; CFR, Coronary flow reserve; HOMA-IR, Homeostasis Model Assessment of Insulin Resistance. Comparisons among the three groups were performed using one-way analysis of variance (ANOVA), with Bonferroni correction for pairwise multiple comparisons. Higher IMR indicates increased microvascular resistance, while lower CFR reflects reduced coronary flow reserve. *P* < 0.05 was considered statistically significant.

### 3.3 Correlation between HOMA-IR and microcirculatory parameters

We further analyzed the correlation between HOMA-IR and microvascular indicators. As shown in Table 3, HOMA-IR was significantly positively correlated with IMR (*r* = 0.436, *P* < 0.001), and negatively correlated with CFR (*r* = –0.408, *P* < 0.001). These findings support a strong association between higher IR and impaired coronary microcirculatory function.

**Table 3:** Correlation between HOMA-IR and microcirculatory function.

Indicator	Correlation coefficient (*r*)	*P*-value	Direction	Clinical interpretation
HOMA-IR vs. IMR	0.436	< 0.001	Positive	Higher HOMA-IR → Higher microvascular resistance
HOMA-IR vs. CFR	−0.408	< 0.001	Negative	Higher HOMA-IR → Lower coronary flow reserve

HOMA-IR, Homeostasis Model Assessment of Insulin Resistance; IMR, index of microcirculatory resistance; CFR, coronary flow reserve. Correlation analysis was performed using Pearson correlation coefficients to evaluate the linear relationship between HOMA-IR and microcirculatory function parameters. A positive correlation indicates a direct relationship, while a negative correlation indicates an inverse relationship. *P* < 0.05 was considered statistically significant.

### 3.4 Logistic regression: predictive value of HOMA-IR for microvascular dysfunction

Based on the established correlation between HOMA-IR and microcirculatory parameters, we further explored whether HOMA-IR serves as an independent predictor of coronary microvascular dysfunction. According to previous literature and clinical guidelines, IMR ≥ 25 and CFR < 2.0 were defined as the thresholds for microcirculatory dysfunction. Univariate analysis revealed that the incidence of dysfunction was significantly higher in the high HOMA-IR group (61.6%) than in the low HOMA-IR group (28.3%) (*χ*^2^ = 21.76, *P* < 0.001). After adjusting for age, sex, LVEF, and Killip classification, multivariate logistic regression showed that high HOMA-IR was an independent risk factor for elevated IMR (*P* = 0.001) and a significant predictor of reduced CFR (*P* = 0.006) (Table 4). These findings suggest that not only is insulin resistance linearly associated with microvascular injury, but its elevation is also an independent risk factor for coronary microcirculatory dysfunction, highlighting its potential value for clinical risk identification.

**Table 4:** Logistic regression analysis of HOMA-IR and coronary microvascular dysfunction.

Dependent variable	Independent variable	Adjusted factors	OR	95% CI	*P*-value
IMR ≥ 25	HOMA-IR (High vs. Low)	Age, sex, LVEF, Killip class	2.84	1.52–5.29	0.001
CFR < 2.0	HOMA-IR (High vs. Low)	Age, sex, LVEF, Killip class	2.41	1.28–4.55	0.006

Logistic regression models were used to evaluate whether high HOMA-IR levels were independently associated with coronary microvascular dysfunction, defined as IMR ≥ 25 or CFR < 2.0. HOMA-IR, Homeostasis Model Assessment of Insulin Resistance; IMR, index of microcirculatory resistance; CFR, coronary flow reserve; LVEF, left ventricular ejection fraction; OR, Odds ratio; CI, Confidence interval. models were adjusted for age, sex, LVEF, and Killip classification at admission. *P* < 0.05 was considered statistically significant.

### 3.5 One-year MACE incidence and Its association with HOMA-IR

To further assess the impact of insulin resistance on long-term clinical outcomes, we conducted a 12-month follow-up for all three HOMA-IR groups to monitor MACE, including all-cause mortality, recurrent myocardial infarction (MI), heart failure hospitalization, and target vessel revascularization. As shown in Table 5, a total of 68 MACE events (22.8%) occurred during follow-up, including 18 deaths, 12 recurrent MIs, 26 heart failure hospitalizations, and 12 revascularizations. The incidence of MACE significantly differed among the three groups (*χ*^2^ = 14.82, *P* = 0.001), with the highest occurrence in the high HOMA-IR group. Kaplan–Meier survival curves demonstrated that patients in the high HOMA-IR group had the lowest event-free survival, and the difference was statistically significant (Log-rank *P* < 0.001). These results indicate that elevated HOMA-IR levels are associated with increased risk of MACE in non-diabetic STEMI patients within 1 year post-PCI and may serve as a potential biomarker for long-term risk stratification.

**Table 5:** One-Year MACE incidence across HOMA-IR groups.

HOMA-IR group	MACE cases (%)	All-cause death	Recurrent MI	HF hospitalization	Revascularization
Low	13 (13.1%)	3	2	5	3
Medium	21 (21.0%)	6	4	9	2
High	32 (32.3%)	9	6	12	5

MACE, major adverse cardiovascular events; MI, myocardial infarction; HF, heart failure; HOMA-IR, Homeostasis Model Assessment of Insulin Resistance. MACE was defined as the first occurrence of any of the following events within 12 months after PCI: all-cause death, recurrent MI, hospitalization for heart failure, or target vessel revascularization. Event data were collected through outpatient visits and/or structured telephone follow-up. Differences in MACE incidence across HOMA-IR groups were analyzed using the chi-square test. *P* < 0.05 was considered statistically significant.

### 3.6 Cox regression analysis: HOMA-IR as an independent predictor of MACE

**Figure 1:**
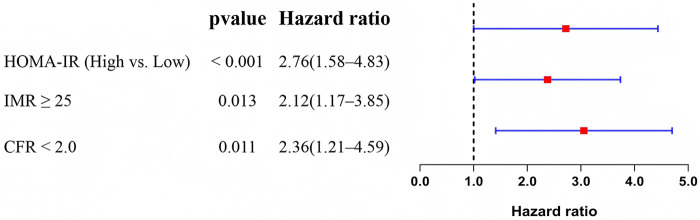
Forest plot of multivariate Cox regression analysis for predictors of 1-year major adverse cardiovascular events (MACE).

To determine whether HOMA-IR is an independent predictor of long-term adverse cardiovascular outcomes, we constructed a multivariate Cox proportional hazards model, adjusting for potential confounders including age, Killip class, LVEF, peak troponin levels, and pre-procedural TIMI flow grade. The analysis revealed that high HOMA-IR was an independent predictor of MACE (HR = 2.76, *P* < 0.001). In addition, IMR ≥ 25 (*P* = 0.013) and CFR < 2.0 (*P* = 0.011) were also independently associated with higher MACE risk (Table 6). These results underscore the prognostic value of HOMA-IR in non-diabetic STEMI patients and emphasize the importance of assessing coronary microvascular function in postoperative risk stratification. The multivariate Cox regression outcomes are visually summarized in Figure 1, showing that elevated HOMA-IR, IMR ≥ 25, and CFR < 2.0 were all independently associated with increased risk of MACE.

**Table 6:** Cox regression analysis: predictors of One-year MACE.

Variable	HR	95% CI	*P*-value
HOMA-IR (High vs. Low)	2.76	1.58–4.83	<0.001
IMR ≥ 25	2.12	1.17–3.85	0.013
CFR < 2.0	2.36	1.21–4.59	0.011

Cox proportional hazards regression models were used to identify independent predictors of 1-year major adverse cardiovascular events (MACE). HR, HAZARD Ratio; CI, confidence interval; HOMA-IR, Homeostasis Model assessment of Insulin Resistance; IMR, index of microcirculatory resistance; CFR, coronary flow reserve. The models were adjusted for potential confounders, including age, Killip class, left ventricular ejection fraction (LVEF), peak troponin level, and pre-procedural TIMI flow grade. *P* < 0.05 was considered statistically significant.

## 4 Discussion

This study demonstrated that in non-diabetic patients with STEMI, elevated insulin IR, as measured by HOMA-IR, was closely associated with coronary microcirculatory dysfunction after PCI. Specifically, higher HOMA-IR levels correlated with increased IMR and decreased CFR. Further analyses confirmed that HOMA-IR was an independent predictor of both microvascular dysfunction and 1-year MACE, indicating that IR has important prognostic implications even in the absence of overt diabetes.

Our findings highlight several potential pathophysiological mechanisms linking IR to microcirculatory dysfunction in non-diabetic STEMI patients. IR is known to reduce endothelial nitric oxide (NO) bioavailability and impair vasodilation, which may lead to coronary microvascular constriction and reperfusion deficits (16). Moreover, IR activates oxidative stress and the NF-κB signaling pathway, promoting the release of proinflammatory cytokines such as IL-6 and TNF-α, thereby exacerbating myocardial apoptosis and microvascular damage (17, 18). Elevated IR also increases levels of plasminogen activator inhibitor-1 (PAI-1) and enhances platelet aggregation, fostering a prothrombotic state that may hinder effective reperfusion and contribute to increased IMR and reduced CFR (19). Additionally, under insulin-resistant conditions, the myocardium shifts its primary energy source from glucose to fatty acids, which increases oxygen consumption and perpetuates a “metabolism-perfusion mismatch,” further impairing reperfusion quality and myocardial recovery (20, 21). Unlike previous studies that primarily focused on diabetic populations, our study is among the first to confirm these mechanisms' impact on microvascular function and clinical outcomes in non-diabetic individuals, underscoring the need to recognize and manage IR as a “hidden metabolic risk”.

This study suggests that HOMA-IR could serve as a potential biomarker for evaluating microvascular function and long-term prognosis in non-diabetic STEMI patients. In clinical practice, patients with elevated IR may benefit from early lifestyle interventions and possibly insulin-sensitizing therapies to improve microvascular perfusion and reduce MACE risk. Future research should focus on prospective interventional trials to evaluate the clinical benefits of targeting IR in STEMI populations. Furthermore, advanced imaging techniques such as cardiac MRI may help clarify the structural basis and evolution of microvascular dysfunction. The development of combined predictive models incorporating IR and other metabolic biomarkers (e.g., SII, NLR) also holds promise for future exploration.

Despite the important clinical implications of our findings, several limitations should be acknowledged. First, this was a single-center retrospective study, which may introduce selection bias and limit the generalizability of the results. Future multicenter prospective investigations are warranted to confirm these observations. Second, insulin resistance was assessed solely using the HOMA-IR index. Although this measure is widely accepted in clinical research, it is subject to assay variability and lacks the ability to track longitudinal changes over time. Third, detailed quantitative coronary angiographic data (e.g., the number of vessels with ≥50% stenosis or lesion complexity) were not systematically available, as the primary focus of this study was microcirculatory physiology rather than epicardial anatomy. Nonetheless, all included patients had angiographically confirmed culprit lesion occlusion consistent with STEMI, ensuring a homogeneous study population. Finally, noninvasive endothelial assessments (e.g., reactive hyperemia index), inflammatory markers (e.g., C-reactive protein, fibrinogen), and metabolic indicators (e.g., waist circumference, waist-to-hip ratio) were not analyzed, and the role of fractional flow reserve (FFR) in assessing stable plaques was beyond the study's scope. Future prospective studies integrating inflammatory, metabolic, endothelial, and angiographic parameters are needed to further elucidate the interplay among insulin resistance, endothelial dysfunction, and coronary microcirculatory impairment.

## 5 Conclusion

In summary, elevated HOMA-IR levels in non-diabetic patients with ST-segment elevation myocardial infarction (STEMI) were significantly associated with coronary microcirculatory dysfunction after PCI and predicted an increased risk of 1-year major adverse cardiovascular events (MACE). HOMA-IR was not only an independent predictor of microvascular impairment but also a strong indicator of long-term adverse prognosis. These findings emphasize the clinical importance of routine assessment of insulin resistance in non-diabetic patients and provide a potential reference for early risk stratification and individualized secondary prevention strategies in STEMI management.

## Data Availability

The original contributions presented in the study are included in the article/Supplementary Material, further inquiries can be directed to the corresponding author/s.
